# Supplementary material for: Antimicrobial and Mycotoxin Reducing Properties of Lactic Acid Bacteria and Their Influence on Blood and Feces Parameters of Newborn Calves
Source: Animals (Basel). 2023 Oct 27;13(21):3345. doi: 10.3390/ani13213345 (PMC10648343; doi:10.3390/ani13213345)
Supplement: Supplementary file 1 [file animals-13-03345-s001.zip › Supplementary File No. 3. Analysis of calves blood parameters.pdf]

Supplementary File No. 3. Analysis of calves' blood parameters

Calves were bled (4 mL) aseptically from jugular vein into vacuum blood tube (BD Vacutainer®, Lithium Heparin Tube, UK) at days 2 and 14 of the experiment before the morning feeding. Samples taken on day 2 were before the treatment started and were used as baseline measurements. All blood samples, prior to analysis (maximum of two hours), until processing, were kept in an ice bath. The blood gas and biochemical profile parameters (hydrogen potential (pH); partial pressure of carbon dioxide (pCO_2, mmHg), partial pressure of oxygen (pO_2, mmHg); oxygen saturation (O_2 saturation, %); sodium (Na, mmol L^{-1}); potassium (K, mmol L^{-1}); ionized calcium (iCa, mmol L^{-1}); total carbon dioxide (tCO_2, mmHg); hematocrit (Hct, %); hemoglobin content (Hgb, g dL^{-1}); glucose (Glu, mmol L^{-1}); lactate (mmol L^{-1}) were analyzed using blood gas analyzer (EPOC, Ottawa, Canada).
