# Supplementary material for: Antimicrobial and Mycotoxin Reducing Properties of Lactic Acid Bacteria and Their Influence on Blood and Feces Parameters of Newborn Calves
Source: Animals (Basel). 2023 Oct 27;13(21):3345. doi: 10.3390/ani13213345 (PMC10648343; doi:10.3390/ani13213345)
Supplement: Supplementary file 1 [file animals-13-03345-s001.zip › Supplementary File No.1. Analysis of mycotoxins biodegradation properties..pdf]

Supplementary File No.1. Analysis of mycotoxins biodegradation properties.

Mycotoxin concentrations reduction were evaluated by UPLC-MS. The separation of mycotoxins was performed on a 100 mm × 2.1 mm i.d., 2.6 μm Kinetex C18 reversed phase analytical column (Phenomenex, USA). The gradient elution system, consisting of 0.1% formic acid in water (mobile phase A) and 100% methanol (mobile phase B) at a flow rate of 0.3 mL min^{-1}, was set as follows: the initial mobile phase of 95% A and 5% B was kept constant for 6 min. From 6 min to 10 min the percentage of phase B was linearly raised to 95% and held constant until 11 min. Then the percentage of phase B was sharply decreased again to 5% over 0.1 min, and was kept at this level until 15 min. The injection volume was 10 μL. The column and sample temperatures were 40 °C and 10 °C, respectively. The analysis was performed as multiple reaction mon- itoring (MRM) in positive and negative ion detection modes. The ESI- MS/MS analysis was performed applying the following parameters: source temperature 500 °C, ion spray voltage 4.50 kV/-3.50 kV, curtain gas nebulizer pressure 30 psi, ion source gas 1 (GS1) 40 psi and ion source gas 2 (GS2) 60 psi. Control of the instrument conditions and data processing were carried out using the Analyst 1.6 software (AB SCIEX, USA). Instrumental parameters for the analysed mycotoxins and validation of the analytical method are fully described by Reinholds et al. [45]. Three parallel replicates of each sample were obtained.
