# Supplementary material for: Antimicrobial and Mycotoxin Reducing Properties of Lactic Acid Bacteria and Their Influence on Blood and Feces Parameters of Newborn Calves
Source: Animals (Basel). 2023 Oct 27;13(21):3345. doi: 10.3390/ani13213345 (PMC10648343; doi:10.3390/ani13213345)
Supplement: Supplementary file 1 [file animals-13-03345-s001.zip › Supplementary File No.2. Analysis of Microbiological Paramaters.pdf]

Supplementary File No.2. Analysis of Microbiological Parameters

For the viable LAB counts evaluation, De Man, Rogosa, and Sharpe (MRS) agar (Oxoid Ltd., Basingstoke, UK) was used. Method in detail described in ISO 15214:1998 [46].

For the total aerobic bacteria and facultative anaerobes evaluation, The plate count agar (Biolife Italiana Srl, Milan, Italy) was used. Method in detail described in ISO 4833-2:2013 [50].

For the the total count of enterobacteria determination, The violet red bile glucose (VRBG) agar (Oxoid Ltd., Basingstoke, UK) was used. Method in detail described in ISO 21528-2:2017 [51].

For the yeast and mold (Y/M) count determination, the dichloran rose bengal chloramphenicol (DRBC) agar (Liofilchem, Milan, Italy) was used. Method in detail described in ISO 21527-2:2008 [52].

The results were expressed as a log_{10} of CFU g^{-1} of a sample.

The calves' fecal samples were collected on days 2 and 14 of life directly from the anus into clean plastic vials immediately after a gloved, lubricated finger was gently passed through the anus to massage the rectal wall and to stimulate rectal evacuation, stored in vials (+4 °C) with a transport medium (Fecal TM enteric Plus, Oxoid, Basingstoke, UK) and analyzed on the same day.
